# Feasibility and Acceptability of an eHealth-Based Physical Activity Coaching Intervention During Pulmonary Rehabilitation for People With Chronic Obstructive Pulmonary Disease: Mixed Methods Study

**DOI:** 10.2196/83783

**Published:** 2026-04-16

**Authors:** Sofia Flora, Ana Sofia Grave, Sara Pimenta, Fátima Baptista, Chris Burtin, Joana Cruz

**Affiliations:** 1 Center for Innovative Care and Health Technology (ciTechCare), Polytechnic of Leiria; Faculty of Human Kinetics, University of Lisbon, Portugal Leiria Portugal; 2 Respiratory Research and Rehabilitation Laboratory (Lab3R), School of Health Sciences (ESSUA), iBiMED – Institute of Biomedicine and Department of Medical Sciences, University of Aveiro, Aveiro, Portugal; C-mo Medical Solutions, Lisboa, Portugal Aveiro Portugal; 3 CIPER, Faculdade de Motricidade Humana, Universidade de Lisboa, Lisbon, Portugal Lisboa Portugal; 4 REVAL - Rehabilitation Research Center, Faculty of Rehabilitation Sciences, Hasselt University, Diepenbeek; BIOMED—Biomedical Research Institute, Hasselt University, Diepenbeek, Belgium Hasselt Belgium; 5 Center for Innovative Care and Health Technology (ciTechCare), Polytechnic of Leiria, Leiria; School of Health Sciences, Polytechnic of Leiria Leiria Portugal

**Keywords:** chronic obstructive pulmonary disease, COPD, eHealth intervention, physical activity, pulmonary rehabilitation

## Abstract

**Background:**

Physical inactivity is a modifiable and significant trait in people with chronic obstructive pulmonary disease (COPD). While traditional exercise-based pulmonary rehabilitation (PR) improves symptoms and exercise tolerance, its impact on physical activity (PA) levels remains limited. Digital health (eHealth) interventions may help address this gap.

**Objective:**

This study aimed to assess the feasibility and acceptability of integrating an eHealth PA coaching intervention into PR for people with COPD.

**Methods:**

Patients enrolled in an outpatient PR program were recruited for a 3-week PA coaching intervention, which used a smart band connected to a mobile patient app and a web application for health care professionals (HCPs). The intervention included PA monitoring (steps per day); weekly goal setting; and app notifications for goal updates, achievement, and motivational messages. Weekly telephone calls supported goal adjustment and identification of PA barriers. The acceptability of the intervention was explored through a patient focus group.

**Results:**

Five patients with COPD (mean 67, SD 9 years; n=4, 80% female; mean predicted forced expiratory volume at 1 second of 49%, SD 23%) participated with 100% retention and adherence to the intervention (daily synchronization). No adverse events or PA barriers were identified. One patient reported an app connection issue that was resolved by restarting the app. Patients found the app easy to use and helpful for their PA awareness and remote monitoring by HCPs. Weekly goal adjustments and contact with an HCP were valued. Limitations regarding the app use included a lack of personalization, goal setting restricted to steps, and occasional step miscounts.

**Conclusions:**

The intervention was feasible and well accepted. Future studies with a larger sample are needed to assess the impact of the intervention on PA outcomes.

## Introduction

Chronic obstructive pulmonary disease (COPD) is a highly prevalent lung disease that imposes a significant burden on health care systems and is a major cause of long-term disability [1]. Physical inactivity is a crucial treatable trait in COPD, as it is related to increased risk of exacerbations, hospitalizations, and mortality [2,3]. Thus, improving physical activity (PA) levels in these patients is a global priority [1].

Pulmonary rehabilitation (PR) is a nonpharmacological and evidence-based intervention known to improve symptomatology and functional performance in patients with COPD [4,5]. Exercise training is considered a cornerstone of PR. Previous studies have found inconsistent results regarding the impact of exercise-based PR on patients’ PA levels. This suggests that behavioral interventions such as PA coaching should be integrated into PR to optimize promotion of an active lifestyle [6-8]. This strategy allows clinicians to detect and intervene early in case of low PA adherence [9].

Therefore, it is crucial to develop strategies to optimize patients’ PA during and after the PR. PA coaching can be supported by information and communication technologies such as eHealth, including mobile apps, as their use is increasingly common in everyday life [10] and technology is embedded into the health care systems [11]. Mobile apps can monitor PA in real time, enabling automated and continuous self-monitoring and feedback [12], facilitating low-burden measurement of activity with acceptable accuracy [13], and providing continuous access to recorded data both longitudinally and in real time [12].

Prior to conducting effectiveness trials, eHealth interventions such as PA coaching must be assessed for feasibility and acceptability to ensure their optimization [14]. Identifying specific barriers is a vital prerequisite for developing robust, personalized, and scalable interventions capable of fostering long-term active lifestyles in patients with COPD. Therefore, this study aimed to evaluate the feasibility and acceptability of an eHealth-supported PA coaching intervention for patients with COPD attending a PR program.

## Methods

### Ethical Considerations

This study was approved by the ethics committee of the institution involved, that is, the Local Health Unit of the Leiria Region (reference 10/2024, acta number 022024/01/16). Participant data were handled in accordance with applicable data protection regulations. Patients who agreed to participate in the study provided written informed consent before any data collection.

### Design

This was a single-group feasibility and acceptability study that investigated a 3-week PA coaching intervention implemented during a PR program. This study is part of the Effectiveness of Physical Activity Coaching using eHealth in patients with COPD (Physical Activity in COPD Using eHealth) study, which aims to assess the effectiveness of a personalized eHealth coaching intervention integrated into PR to increase the PA levels in patients with COPD. This feasibility study was reported in accordance with guidelines for reporting pilot and feasibility studies (adjusted for a single-arm study) [14-16]. Participant data were handled in accordance with applicable data protection regulations. The focus of this study was on the feasibility and acceptability of the intervention, specifically to test the PA goal-setting algorithm and assess the receptivity and acceptance of the intervention, including the digital platform, to identify any necessary adjustments for its implementation in a larger study. For this purpose, 3 weeks were needed for patients to interact with the platform, test its acceptability, and refine the algorithm.

### Participants

Potential participants were recruited from the Local Health Unit of the Leiria Region (Portugal). The participant eligibility criteria are shown in Textbox 1.

**Participant eligibility criteria.**

**Inclusion criteria**

- Aged ≥40 years
- Diagnosis of chronic obstructive pulmonary disease according to Global Initiative for Chronic Obstructive Lung Disease criteria [1]
- Clinically stable (ie, with no exacerbations in the previous month)
- Outpatients attending the pulmonary rehabilitation program at the hospital
- Able to understand and provide informed consent

**Exclusion criteria**

- Simultaneous participation in another physical activity (PA) behavioral modification program
- Not having a smartphone
- Having a clinical condition that could preclude participation in a PA intervention (eg, severe musculoskeletal or neurological disorders)
- Other primary respiratory disease
- Unstable cardiovascular disease and/or history of recent neoplasia (including last treatment) in the previous year

The physician responsible for the PR program identified the eligible participants and briefly explained the study to them. Those interested in participating were then contacted by the researcher via telephone to inform them about the study procedures (data collection, dates, and intervention delivery), confirm their eligibility, and ask about their willingness to participate. Reasons for not participating were recorded. Patients who agreed to participate provided written informed consent before any data collection.

### Procedures

**Figure 1:**
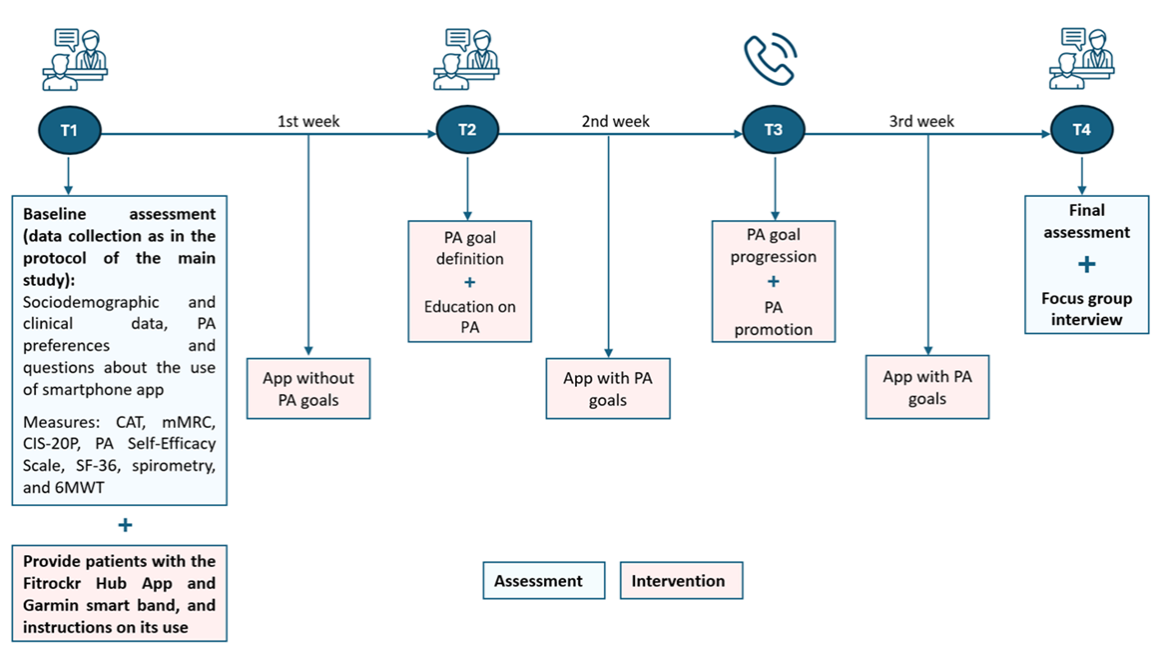
Design of the feasibility study. 6MWT: 6-Minute Walk Test; CAT: COPD Assessment Test; CIS-20P: Portuguese version of the Checklist of Individual Strength; mMRC: modified Medical Research Council; PA: physical activity; SF-36: 36-item Short Form Health Survey.

This study included 4 interaction time points with patients, as described in Figure 1. The assessments took place at baseline (T1) and 3 weeks after, corresponding to the end of the intervention (T4). In the baseline assessment (T1), participants completed a questionnaire with sociodemographic and health-related information (ie, age, sex, educational level, occupational status, smoking history, comorbidities using the Charlson Comorbidity Index [17], exacerbation history, medication, weight, and height), preferred physical activities (eg, walking, swimming, and dancing), and questions about the type and features or apps used on their smartphone. Lung function was assessed through spirometry [18] to further classify patients based on Global Initiative for Chronic Obstructive Lung Disease (GOLD) grades of airflow obstruction severity (GOLD 1: forced expiratory volume at 1 second [FEV_1_]≥80% predicted, GOLD 2: 50%≤FEV_1_<80% predicted, GOLD 3: 30%≤FEV_1_<50% predicted, and GOLD 4: FEV_1_<30% predicted) [1]. GOLD A, B, and E categories were determined according to the patients’ exacerbation history and symptoms [1]. The impact of COPD on daily living, dyspnea, fatigue, exercise tolerance, quality of life, and self-efficacy for PA was also assessed, as described in detail in the Secondary Outcome Measures (Health-Related Outcomes) section.

Then, patients received the PA mobile app paired with a smart band to use during the study period. In the first week, their baseline step count was established for goal setting in the following weeks. In the second time point (T2—end of the first week), patients received education on the importance of PA and recommendations and agreed on the first PA goal set. In the third time point (T3—end of the second week), the researcher reviewed the achievement of the previous PA goal, explored barriers to PA behavior and potential adverse events, and set the next goal with the patient through a telephone call. One week later (T4—end of the third week), patients returned the app and the smart band and were reassessed as in T1. A focus group with patients was also conducted to assess their feedback on the PA coaching intervention, including their experience with new technology; perspectives on the importance of PA promotion interventions; app functionality; and acceptability, barriers, and facilitators of its use. The intervention is described in detail below (refer to the PA Coaching Intervention section)*.*

### Intervention

#### eHealth Platform for PA Coaching

The eHealth platform used in this study was the *Fitrockr Hub App* (Fitrockr Health Solutions). This eHealth platform enables tailoring PA coaching to each patient by monitoring patients’ adherence to predefined PA goals and enabling individualized notifications.

The Fitrockr Hub App platform comprises 2 applications: a web application for HCPs and a mobile app for patients. The mobile app synchronizes with a Garmin smart band (Garmin Vivosmart 4; Garmin), which allows for PA monitoring (ie, steps, distance, and calories), access to PA history (in days), daily PA goals (defined in the web application), and notifications on the patient’s performance based on the fulfillment of their personal goals. The web application for the HCPs allows them to monitor patients’ daily PA to define and/or change individualized goals, which will then appear on the home page of the mobile app. Through the web page, HCPs can also insert new patients, prescribe personalized PA goals for each patient (ie, steps), monitor their daily PA activity (eg, observe how many steps take on a given day), check whether patients met their prescribed goals, and send personalized notifications. The data can be exported to Excel files for further analysis.

#### PA Coaching Intervention

In this study, the principal researcher assumed the role of the HCP responsible for PA coaching, with exclusive access to the PA platform and responsibility for delivering the intervention. Hospital HCPs were responsible for the delivery of the PR program, which all participants attended as usual, with the PA coaching intervention provided as an additional component.

The PR program was delivered in accordance with the European Respiratory Society’s and American Thoracic Society’s recommendations [5] and had a duration of 8 weeks, as recommended. Participants were not all at the same stage of the PR program at the time of enrollment in the study, but the same protocol (Figure 1) was applied to all participants.

Patients were asked to wear the smart band connected to the Fitrockr Hub App every day for 1 week to set their baseline steps (T1). As the mobile app does not synchronize automatically with the smart band, patients were asked to manually synchronize them twice a day (ie, upon awakening and before retiring for the night), every day, by clicking a button in the app.

**Figure 2:**
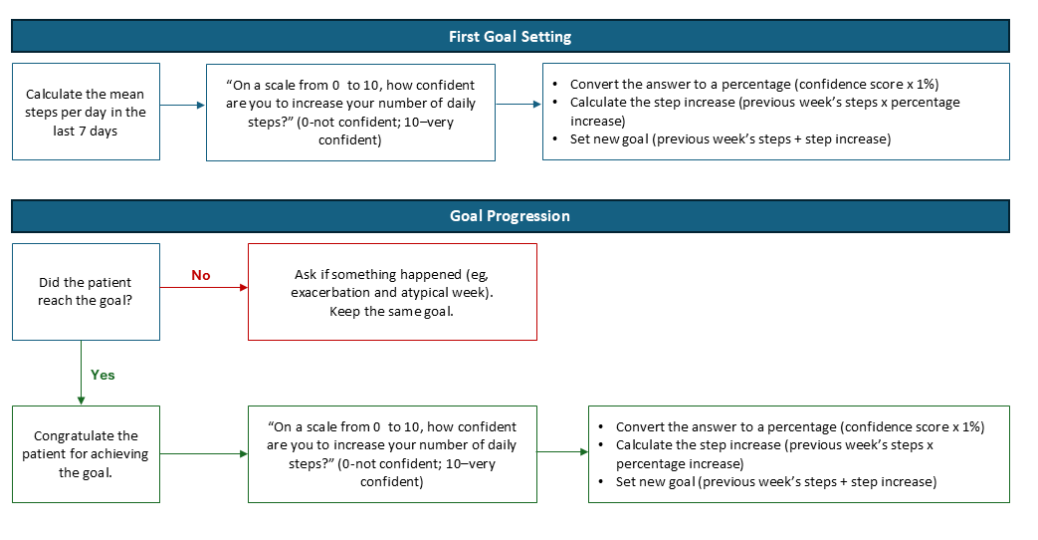
Algorithm for goal setting and progression of physical activity coaching.

After the first week (T2), patients received information about the importance and recommendations of PA, and the first PA goal was set (in person). The personalization of the goal was based on an algorithm (Figure 2) that considered the amount of PA performed in the previous week (ie, the average number of daily steps in the last 7 days) and patients’ confidence in changing the goal. The “confidence” level was then translated into a percentage increase from the previous goal, and the adjusted goal was then displayed in the Fitrockr Hub App. To avoid the risk of injuries and to ensure tolerability, the weekly increase in PA goals did not exceed 10% [19]. During the week, patients received an automatic notification every day at 6 PM about their goal achievements, providing them with the opportunity to increase their PA to reach their daily goal if needed. During the intervention, patients received sporadic personalized notifications sent manually by the researcher (eg, notifications about bad weather suggesting indoor activities and incentive messages). At the end of the second week (T3), patients received a telephone call to check their PA progression. If patients did not achieve their goals in the previous week, the researcher asked them to identify potential barriers to PA improvement and discussed possible strategies to overcome them (examples are provided in Multimedia Appendix 1). Then, the goal for the next week was set according to the algorithm presented in Figure 2. During the intervention, patients were allowed to contact the researcher by telephone to receive technical support, if needed.

Reasons for not progressing the goals as well as possible adverse events and technical issues related to app functioning were recorded. The diary of telephone calls and goal progression was recorded and is provided in Multimedia Appendix 2.

As PA is a complex behavior [20], the intervention included several behavior change techniques (BCTs), according to the BCT Taxonomy v1 [21], including goals and planning, feedback and monitoring, natural consequences, repetition and substitution, comparison of outcomes, and self-belief. More information on how these BCTs were implemented is provided in Multimedia Appendix 3.

### Outcomes

#### Primary Outcomes

##### Feasibility

Feasibility was defined as the extent to which the intervention can be successfully implemented within a given setting or larger study [22]. Feasibility was evaluated through the following parameters assessed during the intervention:

Feasibility of the study design:

- Recruitment rate (%)=(number of recruited patients÷number of eligible patients)×100
- Study retention (%)=percentage of patients who completed the intervention and data collection (The expected recruitment rate and study retention were 80%, at least [15])
- Dropout rate (and its reasons)=(number of dropouts÷number of patients recruited)×100

Feasibility of the intervention:

- Adherence to intervention (%) was assessed by the number of days patients had synchronized the app with the smart band throughout the intervention, regardless of whether they achieved the goal.
- Intervention safety=adverse events and/or unplanned health care use (self-reported)
- Technical issues about app functioning (self-reported)
- Duration and content of the telephone calls

##### Acceptability

Acceptability was defined as the extent to which patients receiving the intervention considered it appropriate to promote PA during PR [23]. Acceptability was assessed through a focus group with a semistructured guide and audio-recorded for posterior transcription and analysis. The focus group guide is provided in Multimedia Appendix 4. No field notes were made during the interview. The focus group covered the following topics: (1) feedback about the intervention—questions about patients’ experience with the intervention, specifically the importance of this type of intervention to improve their PA levels, barriers and facilitators to the use of mobile apps to promote PA, goal setting and progression, notifications, and HCPs’ contact; and (2) feedback about the functioning of the app—questions about barriers and facilitators of app use: the experience of interacting with the smartphone app and the smart band, as well as the degree of technological complexity.

#### Secondary Outcome Measures (Health-Related Outcomes)

Given the short duration of the intervention (3 weeks), no changes in health-related outcomes were expected. However, these measures were included in assessment moments to replicate the procedures of the subsequent randomized controlled trial data collection processes. Functional exercise capacity was measured using the 6-Minute Walk Test [24], and predicted values were computed using the equation developed for Portuguese adults [25]. The test was conducted in a 30-meter corridor according to guidelines from the European Respiratory Society and American Thoracic Society [26]. Vital signs were measured before and after the test [27], and symptom scores for dyspnea and fatigue were assessed using the modified Borg scale [28]. The impact of COPD on daily living was assessed using the COPD Assessment Test [1], dyspnea using the modified Medical Research Council Dyspnea Scale [29], quality of life using the Medical Outcomes Study 36-item Short Form Health Survey version 2 [30,31], and self-efficacy for PA using an adapted version of the Physical Exercise Self-Efficacy Scale (adapted with the authors’ permission) [32]. Fatigue was measured with the Checklist of Individual Strength and categorized into 3 categories (normal fatigue: score ≤26, mild fatigue: score between 27 and 35, and severe fatigue: score ≥36) [33].

### Data Analysis

Descriptive statistics were used to characterize the sample using SPSS Statistics (version 28.0; IBM Corp). Continuous variables were summarized as mean (SD) when normally distributed and as median (IQR) when normality assumptions were not met. Categorical variables were described using absolute frequencies (n) and percentages (%).

Data collected through the focus group were analyzed using a qualitative methodological approach, namely, thematic analysis [34]. Participants were anonymized using alphanumeric codes (P1, P2, etc) during data analysis. Themes and categories were developed in an Excel spreadsheet, enabling the identification of the main topics discussed by the participants. The analysis was primarily conducted independently by 2 researchers. Subsequently, both researchers met to systematically compare codes and themes and to reach consensus. Themes were generated and analyzed question by question, following the focus group guide. Once no new categories were identified, the information was organized into a descriptive framework [34].

### Researchers’ Reflexivity

The interviews were conducted by 2 female physiotherapists with a master’s degree in the respiratory field and experience in PA assessment and promotion. Both researchers made efforts to ensure neutrality throughout the analysis process, complying with established qualitative research rigor guidelines. The research team also included a senior member with experience in COPD management, PA promotion, and qualitative research.

## Results

All patients were recruited in March 2024, and the follow-up lasted until May 2024. A total of 6 patients participating in an outpatient PR program were eligible for recruitment; however, 1 patient refused to participate due to lack of interest; therefore, the final sample consisted of 5 patients.

### Patients’ Characteristics

Patients had a mean age of 67.0 (SD 8.9) years and a mean predicted FEV_1_ of 49% (SD 22.8%), indicating a population with moderate to severe airflow limitation. The sample was predominantly female (n=4, 80%), with most patients classified as GOLD E (n=4, 80%), suggesting a high risk of exacerbations and symptom burden. Regarding educational level, 60% (n=3) completed 10 to 12 years of formal education, and all patients were retired (n=5, 100%). The mean COPD Assessment Test score was 13.0 (SD 9.0). Fatigue levels, assessed using the Portuguese version of the Checklist of Individual Strength, showed a mean score of 78.4(SD 28.9). Dyspnea severity, measured by the modified Medical Research Council Dyspnea Scale, had a median score of 3.0 (IQR 0.5-3.5). On average, each assessment session lasted approximately 40 minutes. Characteristics of participants are presented in Table 1.

**Table 1:** Participants’ sociodemographic and clinical characteristics (N=5).

Characteristics			Values
Age (years), mean (SD)			67.0 (8.9)
FEV_1_^a^ % predicted, mean (SD)			49.0 (22.8)
BMI (kg/m^2^), mean (SD)			26.3 (8.7)
**Sex, n (%)**			
	Female	4 (80)	
	Male	1 (20)	
**Education level, n (%)**			
	5-9 years	1 (20)	
	10-12 years	3 (60)	
	Higher education	1 (20)	
**GOLD ABE^b^ classification, n (%)**			
	GOLD A	1 (20)	
	GOLD E	4 (80)	
**GOLD FEV_1_ classification, n (%)**			
	GOLD 1	1 (20)	
	GOLD 2	1 (20)	
	GOLD 3	2 (40)	
	GOLD 4	1 (20)	
**CCI^c^, n (%)**			
	Mild	1 (20)	
	Moderate	4 (80)	
CAT^d^ total score, mean (SD)			13.0 (9.0)
CIS20-P^e^ total score, mean (SD)			78.4 (28.9)
**CIS20-P subjective fatigue, n (%)**			
	Normal fatigue	1 (20)	
	Mild fatigue	2 (40)	
	Severe fatigue	2 (40)	
mMRC^f^, median (IQR)			3.0 (0.5-3.5)
6MWD^g^ (meters), mean (SD)			419.0 (102.0)
6MWD (% predicted), mean (SD)			88 (23)

^a^FEV_1_: forced expiratory volume at 1 second.

^b^GOLD: Global Initiative for Obstructive Lung Disease Groups A, B, E.

^c^CCI: Charlson Comorbidity Index.

^d^CAT: COPD Assessment Test.

^e^CIS20-P: Portuguese version of the Checklist of Individual Strength.

^f^mMRC: modified Medical Research Council.

^g^6MWD: 6-Minute Walk Distance.

Regarding the experience with new technologies, all patients used smartphones, performing various tasks on them, such as browsing the internet and listening to music (n=5, 100%); sending or receiving SMS text messages (n=4, 80%); taking photos, playing games, and using social media (n=3, 60%); and using GPS (n=2, 40%).

### Feasibility of the Study Design

The recruitment rate was 83% (5/6) with 100% retention and no dropouts.

### Feasibility of the Intervention

During the intervention, no adverse events or unplanned health care use were reported. Only 1 technical issue with the app was reported, involving difficulties connecting to the app; the researcher restarted the app, and the problem was resolved. Adherence was 100% (5/5), with patients syncing the mobile app daily. The call for goal progression (T2) lasted an average of 10 (SD 3.13) minutes. The summary of goal progression and diary of telephone calls (to report technical issues from the app, goal setting, and PA promotion) was recorded, and it can be found in Multimedia Appendix 2.

### Acceptability

1. The focus group lasted 1 hour and 6 minutes, and the several themes were identified.

Value of the PA coaching intervention using eHealth and its impact on PA levels—all patients considered this type of intervention an added value, both for themselves to increase their perception of PA and for health care professionals (HCPs) to help them monitor patients’ PA levels:

I think this is important also for doctors to see our capabilities and solve our problems accordingly...it provides guidance [on PA] to the doctor.

Patient #5

I think this helps us to have a better idea of our effort and to know if we need to walk more….

Patient #2

2. Barriers and facilitators of the use of Fitrockr Hub App to promote PA—the focus only on step counts to define PA goals, the lack of personalization (excluding PA activities other than walking), occasionally step miscounts (through the smart band), and not having the app for free in the real context were considered barriers. Participants identified a user-friendly layout, acceptable time demands, and the prescription of PA goals as facilitators:

This app should have more options than just walking...it is reductive in this way.

Patient #2

In my personal opinion, I think it’s extremely important [PA goals]; it encourages us to exercise more because we have that goal, and if we focus on a goal, we’ll likely try to achieve it every day.

Patient #1

3. Goal setting, progression, PA monitoring, and promotion—patients considered goal setting to be a collaborative and personalized process, highlighting the importance of negotiated PA goals with their HCPs. Patients considered the adjustment of their PA goals (based on performance and willingness to increase) adequate and emphasized the identification of barriers to PA and strategies to overcome them every week as being beneficial for PA engagement. The main barriers to not reaching the PA goals by patients were bad weather, exacerbations or worsening of symptoms, and variability in response to PA. The importance of maintaining contact with HCPs during the intervention, either via video call or telephone, was also highlighted by all patients:

Each patient has their own PA goal, and this is important because people have different health conditions, not everyone has de same degree of severity so you cannot set the same goals for everyone.

Patient #2

(the revision of PA goals) should be weekly because if there is any correction or adjustment needed, it can be done (right away).

Patient #4

It was great to have a possibility to contact our healthcare professional.

Patient #3

In week 2, I have the goal of 8000 steps/day but this coincided with cold weather and I did not have achieve the goal because I was not going leave the house with bad weather...

Patient #2

I think is a good option to talk by telephone calls...[in that way,] the colleagues that live far away do not need to come on purpose if they can talk by telephone.

Patient #4

By videocall is better...the communication is done a lot through gestures, eyes, voice...and you can even get a better sense of whether the person is more or less engaged based on their facial expression.

Patient #2

## Discussion

### Main Findings

Overall, this PA coaching intervention using eHealth appears to be feasible and well accepted by patients. Patients’ feedback about the intervention was positive, as they considered the app easy to use and valuable for PA self-management and remote monitoring by HCPs. Patients underlined the importance of weekly goal adjustments, frequent app notifications, and weekly contact with HCPs through telephone or video calls. However, patients identified some limitations regarding the use of the app, including a lack of personalization, the exclusive focus on step counts to define PA goals, and occasional step miscounts.

Despite a shorter intervention period compared with similar eHealth-based PA interventions in COPD [35,36], this study achieved high recruitment and retention rates (>80%) supporting the feasibility of this study design [15]. These rates are comparable to or higher than those reported in previous feasibility studies, suggesting that integrating PA coaching alongside PR is practicable within this clinical context. However, these findings should be interpreted with caution due to the small and relatively homogeneous sample, which was predominantly female, retired, and familiar with smartphone use. Therefore, the generalizability of these results is limited. Consistent with previous literature, future studies should recruit more diverse samples, including individuals with lower digital literacy [37], varying levels of motivation for PA [38], and higher multimorbidity burden [39], as these factors are known to influence engagement with eHealth interventions.

Regarding intervention feasibility, no major technical or PA barriers were identified during implementation. This contrasts with previous reports highlighting poor digital literacy and technical challenges as key barriers to eHealth adoption in COPD [40] and may be partly explained by the short intervention duration and the provision of technical support. Over time, factors such as comorbidities, environmental challenges, and psychological barriers (eg, fear of breathlessness) can become more pronounced, potentially impairing adherence to the intervention [41].

Regarding the feasibility of PA goal-setting algorithm, although participants did not consistently achieve their weekly goals, they reported that the algorithm was appropriate, with a period of revision and incremental increases being acceptable. These findings align with previous literature recommending weekly goal adjustments, with step count increases ranging from 5% to 15%, tailored to individual tolerance and adherence levels [42-44].

The intervention was proven to be acceptable—patients demonstrated full adherence by synchronizing the smart band with the app daily, regardless of goal achievement. Most patients found the PA app easy to use and well integrated into daily life, supporting evidence that the simplicity and usability of technology can be more decisive than personalization for engagement, as technical issues often lead to participant’s dropout [35]. Patients also highly valued the weekly contact with HCPs, which aligns with evidence highlighting patient-professional interaction as a key facilitator of PA promotion [45], particularly in eHealth interventions [40]. Consistent with the literature, personalized goal setting, tailored to individual needs, and feedback notifications were identified as essential components of these interventions [46,47]. However, important limitations were identified, including the exclusive use of step-based goals and limited app personalization, which may reduce patients’ motivation, engagement with the intervention, and sustained behavior change. While the eHealth platform could not be modified, as it was already developed and PA coaching may not have been its primary focus, future studies should consider greater personalization to better meet patients’ needs. Patients reported PA barriers consistent with the literature, particularly health-related issues (eg, symptom exacerbations), individual variability, and environmental factors such as weather [48]. Addressing these barriers is essential for developing effective and individualized PA interventions. In this study, several evidence-based behavioral strategies, such as goal setting, feedback and monitoring, repetition, substitution, and self-belief, were applied and have since been shown to improve PA in individuals with COPD by supporting barrier identification and problem-solving [49-51].

The findings of this feasibility study suggest that the goal-setting algorithm is appropriate for patients with COPD. Weekly contact between HCPs and patients to establish PA goals, identify potential barriers and corresponding strategies to overcome them, and provide feedback on performance emerged as essential components to retain in future larger-scale studies.

### Limitations

Although PA coaching using eHealth may represent a promising approach for self-managing COPD, the findings of this study should be interpreted with caution due to the small sample size and the predominance of participants with prior digital literacy. This limits the generalizability of the results, as previous literature has indicated that individuals with greater interest and experience in technology are more likely to benefit from eHealth interventions [52]. Given the short duration of the intervention, significant changes in PA behavior were not expected. Instead, collecting health-related outcome measures at baseline and the end of the study primarily served to assess the feasibility of data collection procedures and inform refinements for future large-scale trials.

### Conclusions

This feasibility and acceptability study showed that the PA coaching intervention using eHealth is feasible and well accepted by the patients. However, some barriers still need to be addressed in future studies to ensure adherence to the intervention. Regarding feasibility, while the remote monitoring procedures were successful, future research must prioritize the robustness of the digital infrastructure to eliminate technical errors and data discrepancies, which can undermine clinical reliability. To ensure acceptability and long-term adherence, it is critical that future interventions move toward a more comprehensive model of personalized coaching. This approach should be tailored to each patient’s unique clinical characteristics and PA goals.

## Appendix

Multimedia Appendix 1 Suggestions on how to overcome barriers to physical activity.

Multimedia Appendix 2 Records of goal progression and diary of the weekly phone calls.

Multimedia Appendix 3 Behavior change techniques included in the intervention.

Multimedia Appendix 4 Focus group guide.

## Abbreviations

BCT: behavior change technique
COPD: chronic obstructive pulmonary disease
FEV1: forced expiratory volume at 1 second
GOLD: Global Initiative for Chronic Obstructive Lung Disease
HCP: health care professional
PA: physical activity
PR: pulmonary rehabilitation
