# Supplementary material for: Feasibility and Acceptability of an eHealth-Based Physical Activity Coaching Intervention During Pulmonary Rehabilitation for People With Chronic Obstructive Pulmonary Disease: Mixed Methods Study
Source: JMIR Form Res. 2026 Apr 16;10:e83783. doi: 10.2196/83783 (PMC13133593; doi:10.2196/83783)
Supplement: Multimedia Appendix 1 [file formative_v10i1e83783_app1.docx]

Multimedia Appendix 1 – Suggestions on how to overcome barriers to physical activity.

| **Barriers** | **Suggestions to overcome the barriers** |
| --- | --- |
| **Lack of time** | ▪ Identifying periods of available time during the day in which physical activity can be performed at least three times per week.  ▪ Adding physical activity to patients’ routine (e.g., walking with a friend, relative, or dog; performing PA while watching television…) |
| **Social**  **influence** | ▪ Ask patients to explain to friends and relatives the interest in physical activity to receive support on that behaviour.  ▪ Ask patients to invite friends and relatives to perform physical activity together. Plan social interactions that involve physical activity. |
| **Lack of**  **energy** | ▪ Organise moments of physical activity in periods when patients feel more energy. |
| **Lack of**  **motivation** | ▪ Include the physical activity activities in the calendar, with the date and time, to prevent patients from giving up.  ▪ Suggest that the patients take part in groups of physical activity which follow pre-established frequency |
| **Fear of**  **injuries** | ▪ Instruct the patients to avoid injuries by warming up and stretching of the muscles to be worked out.  ▪ Teach patients how to exercise adequately, considering their health condition. |
| **Lack of**  **resources** | ▪ Suggest activities that require minimal resources, such as walking or walking down/upstairs. |
| **Climatic**  **conditions** | ▪ Prepare a list of activities the patients can perform in bad weather situations (e.g., dancing, cycle-ergometer, active exercises, calisthenics, walking upstairs). |

*Reference: ZuWallack, C.B.R., Exercise Training in Pulmonary Rehabilitation, in Textbook of Pulmonary Rehabilitation, A.E.H. Enrico Clini, Fabio Pitta, Thierry Troosters, Editor. 2018, Springer Cham.*
