# Supplementary material for: Feasibility and Acceptability of an eHealth-Based Physical Activity Coaching Intervention During Pulmonary Rehabilitation for People With Chronic Obstructive Pulmonary Disease: Mixed Methods Study
Source: JMIR Form Res. 2026 Apr 16;10:e83783. doi: 10.2196/83783 (PMC13133593; doi:10.2196/83783)
Supplement: Multimedia Appendix 2 [file formative_v10i1e83783_app2.docx]

Multimedia Appendix 2 – Records of goal progression and diary of the weekly phone calls.

| **First goal setting (T2)** | | | | | | |
| --- | --- | --- | --- | --- | --- | --- |
| **ID** | **Average steps in previous week** | | **0-10 How confident?** | **Additional steps** | **New goal** | **Observations** |
| **P1** | 6205 |  | 0 | 0 | 6200 | Patient felt fear of progress and preferred to maintain the goal to reach it 2 consecutive times. |
| **P2** | 8055 |  | 5 | 403 | 8500 | The patient wanted to round up the goal.  One phone call to technical support (duration 5.05 min). |
| **P3** | 5221 |  | 10 | 522 | 5740 | Patient reported difficulties connecting the app, which was solved by restarting the app. |
| **P4** | 5832 |  | 6 | 349 | 6181 |  |
| **P5** | 9597 |  | 1 | 0 | 9600 |  |
| **Goal progression (T3) (through a telephone call)** | | | | | | |
| **ID** | **Previous goal** | **Steps given in the previous week** | **0-10 How confident?** | **Additional steps** | **New goal** | **Observations** |
| **P1** | 6200***** | 6619 | 0 | 0 | 6500 | Patient did not want to increase the goal (despite having hit it 2 times in a row). Researcher proposed an intermediated value. Patient agreed.  Call duration: 10.35min |
| **P2** | 8500 | 7511 | 0 | 0 | 8500 | As didn't reach the goal, patient preferred to keep it.  Call duration: 14.40min |
| **P3** | 5740 | 3319 | 0 | 523 | 3320 | Call duration: 6.08min |
| **P4** | 6181***** | 8795 | 3 | 205 | 6181 | Call duration: 8.25min |
| **P5** | 9600***** | 9594 | 4 | 384 | 9597 | Call duration: 11.14 min |
| **Goal progression (T4)** | | | | | | |
| **ID** | **Previous goal** | **Steps given in the previous week** | **0-10 How confident?** | **Additional steps** | **New goal** | **Observations** |
| **P1** | 6500 | 5917 |  |  |  |  |
| **P2** | 8500***** | 9205 |  |  |  |  |
| **P3** | 3320 | 2989 |  |  |  |  |
| **P4** | 6181 | 4135 |  |  |  |  |
| **P5** | 9597 | 7113 |  |  |  |  |

***Legend: * - Goal achieved.***
