# Supplementary material for: Feasibility and Acceptability of an eHealth-Based Physical Activity Coaching Intervention During Pulmonary Rehabilitation for People With Chronic Obstructive Pulmonary Disease: Mixed Methods Study
Source: JMIR Form Res. 2026 Apr 16;10:e83783. doi: 10.2196/83783 (PMC13133593; doi:10.2196/83783)
Supplement: Multimedia Appendix 3 [file formative_v10i1e83783_app3.docx]

Multimedia Appendix 3 - Behaviour change techniques included in the intervention.

| **Cluster** | **Behaviour Change Technique** | **Definition** | **Intervention** |
| --- | --- | --- | --- |
| **1. Goals and planning** | 1.1. Goal setting (behaviour) | Set or agree on a goal defined in terms of the behaviour to be achieved. | Negotiated PA goals between the healthcare professional and the patient on a weekly basis. |
|  | 1.2. Problem- solving | Analyse, or prompt the person to analyse factors influencing the behaviour and generate or select strategies that include overcoming barriers and/or increasing facilitators. | Identification of barriers to PA practice and possible strategies to overcome them during the telephone calls. |
|  | 1.5. Review behaviour goals | Review behaviour goal(s) jointly with the person and consider modifying goal(s) or behaviour change strategy in light of the achievement. | Weekly goal revision/progression by the healthcare professional and the patient. |
| **2. Feedback and monitoring** | 2.2. Feedback on behaviour | Monitor and provide informative or evaluative feedback on the performance of the behaviour. | Patients receive feedback about their PA performance during the intervention through the app. |
|  | 2.3. Self-monitoring of behaviour | Establish a method for the person to monitor and record their behaviour(s) as part of a behaviour change strategy. | Patients synchronize the app with the smartband and observe their PA history (steps, calories and distance) on a daily basis. |
| **5. Natural consequences** | 5.1. Information about health consequences | Provide information about the health consequences of performing the behaviour. | Patients are informed about the health benefits of PA practice during the first goal setting moment. |
|  | 5.6. Information about emotional consequences | Provide information about the emotional consequences of performing the behaviour. | Patients are informed on the health benefits of PA practice during the first goal-setting moment, including depression and anxiety. |
| **8. Repetition and substitution** | 8.2. Behaviour substitution | Prompt substitution of the unwanted behaviour with a wanted or neutral behaviour. | During intervention, the healthcare professional suggests the substitution of sedentary activities for more active activities of daily living, such as going for a walk instead of sitting while watching television. |
|  | 8.7. Graded tasks | Set easy-to-perform tasks, making them increasingly difficult but achievable until behaviour is performed. | The PA goals negotiated between the healthcare professional and the patient are based on their PA performance in the previous week, being gradually increased according to patient’s confidence and willingness to progress. |
| **9. Comparison of outcomes** | 9.1. Credible source | Present verbal or visual communication from a credible source in favour of a behaviour. | During education on PA moment, the healthcare professional highlights the benefits of PA practice based on scientific evidence, adapting the speech for a better understanding by the patient. |
| **15. Self-belief** | 15.1. Verbal persuasion about capability | Tell the person they can successfully perform the wanted behaviour, arguing against self-doubts and asserting that they can and will succeed. | The healthcare professional reassured the patients that they could successfully increase their PA levels despite their disease. |

***Reference****: Michie, S., et al., The Behavior Change Technique Taxonomy (v1) of 93 Hierarchically Clustered Techniques: Building an International Consensus for the Reporting of Behavior Change Interventions. Annals of Behavioral Medicine, 2013.* ***46****(1): p. 81-95.*
