# Supplementary material for: Feasibility and Acceptability of an eHealth-Based Physical Activity Coaching Intervention During Pulmonary Rehabilitation for People With Chronic Obstructive Pulmonary Disease: Mixed Methods Study
Source: JMIR Form Res. 2026 Apr 16;10:e83783. doi: 10.2196/83783 (PMC13133593; doi:10.2196/83783)
Supplement: Multimedia Appendix 4 [file formative_v10i1e83783_app4.docx]

**Multimedia Appendix 4 – Focus group guide.**

Thank you for agreeing to participate in this meeting regarding the physical activity (PA) promotion intervention through a mobile application (app) for chronic obstructive pulmonary disease (COPD), which is part of the doctoral research project conducted by the investigator. For further data analysis, the conversation will be recorded with your permission. We assure you that this information will be used solely for research purposes and that your privacy will always be safeguarded.

During our conversation, several topics and questions will be presented on which I would like you to share your thoughts. I am available to repeat any question that you may not have understood and to clarify any doubts that may arise. The participation of everyone is important for the group discussion and the sharing of ideas. There are no right or wrong answers. I kindly ask you to speak one at a time to ensure the quality of the recording. To ensure that the recording accurately reflects your opinions, I also ask that you refrain from gestures of agreement or disagreement, such as nodding your head, and that you share your thoughts, preferably in words. Shall we proceed with the recording?

**Topic “Experience level with new technologies”:**

1. How would you consider your experience with new technologies, particularly with smartphones and mobile applications?

**Topic “To know the importance of intervention to promote patients’ PA levels”:**

1. What is your overall level of satisfaction with the PA promotion intervention, using a mobile app?
2. How has the PA promotion intervention influenced your daily life? And your daily PA levels?

**Topic “Utility/Usefulness of the *Fitrockr Hub App* for promoting PA”:**

1. What is your opinion on the Fitrockr Hub App?
   1. What did you consider essential in the app?
   2. What did you consider secondary or less relevant in the app?
2. How do you perceive the use of a smartband throughout the day, which works together with the app to provide information on PA (i.e., number of steps)?

**Topic “To identify the relevance of the timing of the intervention (along with the pulmonary rehabilitation programme)”:**

1. What is your opinion on integrating the PA promotion intervention into pulmonary rehabilitation programmes?

**Topic “Barriers and facilitators of use of a mobile app for promoting PA (including smartband)”:**

1. What would encourage you to use this app to promote your daily PA? *(Note: for example, low cost, ease of use, ability to communicate with healthcare professionals...)*
2. What would discourage you from using the app?

**Topic “Degree of technology complexity”:**

1. How would you assess the app in terms of complexity or ease of use?

**Topic “Opinion about daily interaction with the app”:**

1. How would you rate your daily interaction with the app?
   1. Was the interaction with the mobile app appropriate to your availability? In terms of time and complexity?

**Topic “Technical problems with the app”:**

1. How would you rate the technical performance of the mobile app?

**Topic “To identify the patients’ feedback regarding goal setting and progression”:**

1. The app allowed the prescription of personalised PA goals and sent you notifications about your performance. What is your opinion on the establishment of PA goals?
2. What is your opinion on how the goals were defined? (e.g., negotiation between patient/healthcare professional; appropriate to expectations/capabilities...)
3. What is your opinion on the progression of the goals? (e.g., frequency and way of review…)
4. The mobile app can also inform the user whether they have achieved their goals or not. What are your thoughts on this?

**Topic “To identify the patients’ preferences about notifications”:**

1. What is your opinion on the notifications, in terms of content and frequency?
2. What impact do you think the notifications had on your PA practice?

**Topic “To identify patients’ preferences about contact with health professionals”:**

1. What is your level of satisfaction with the phone follow-up, and why?
2. How valuable and appropriate were the frequency, duration, and content of the calls?

**20.** Would you like to share any additional information with us that was not addressed in the previous questions?
